# Major historical dietary changes are reflected in the dental microbiome of ancient skeletons

**DOI:** 10.1186/2041-2223-4-10

**Published:** 2013-05-17

**Authors:** Antti Sajantila

**Affiliations:** 1 Department of Forensic Medicine, Hjelt Institute, University of Helsinki, Kytosuontie 11, Helsinki, 00300, Finland

## Abstract

The post-industrial lifestyle has many disadvantageous effects on our health. One of the factors is modern nutrition, which has been associated with epidemic burdens, such as obesity and cardiovascular diseases. At least two major shifts have occurred in the nutritional history of humans: the use of carbohydrate-rich diets which were adopted around 10,000 years BP due to Neolithic farming, and later the influence of industrially processed flour and white sugar after the industrial revolution in the 1850s. In a recent paper in *Nature Genetics* Adler *et al*. used a novel approach to see how these dietary changes affected the oral microbiome by analyzing the ancient microbial DNA in the calcified dental plaque from 34 early European skeletons.

##  

The post-industrial lifestyle has many disadvantageous effects on our health. One of the factors is modern nutrition, which has been associated with epidemic burdens, such as obesity and cardiovascular diseases. At least two major shifts have occurred in the nutritional history of humans: the use of carbohydrate-rich diets which were adopted around 10,000 years BP due to Neolithic farming, and later the influence of industrially processed flour and white sugar after the industrial revolution in the 1850s. In a recent paper in *Nature Genetics* Adler *et al.*[1] used a novel approach to see how these dietary changes affected the oral microbiome by analyzing the ancient microbial DNA in the calcified dental plaque from 34 early European skeletons. The skeletons included both sexes, ranging in age from <20- to >60-years old and dating from the Mesolithic to Medieval times. The authors sampled supra- and subgingival calculus and created PCR amplicon libraries of the 16S rRNA gene and targeted for three hypervariable regions (V1, V3 and V6). In addition, they sequenced the currently known oral pathogens, *Streptococcus mutans* and *Porphyromonas gingivalis* in those samples*.* In order to control for the background environmental bacterial contribution and to compare the data to modern sequences, the authors sequenced in an analogous manner the bacterial DNA from within the same teeth, and calculus and plaque from modern teeth, respectively.

First, the authors needed to show that their data represent authentic microbial sequences. They could show that the calculus from ancient teeth was dominated by Firmicutes bacteria, which were found in a frequency comparable to those found in modern oral samples, and in the Human Oral Microbiome Database (HOMD), whereas the control samples were dominated by Proteobacteria, typically found in clean-room environment and non-template controls. Further data on the authenticity of the microbes in the ancient calculus was indicated by the β-diversity and cluster analysis of the V1, V3 and V6 sequences, which showed more similarity among the ancient and modern calculus, plaque and saliva than among the ancient and environmental samples.

The most interesting part of the study is the analysis of the temporal microbial change in the dental calculus from hunter-gatherers (HGs) to modern humans (MHs). The authors show that the HGs’ dental calculus had fewer bacterial taxa related to caries or periodontal diseases, which is associated with the increased use of soft carbohydrate foods in the Neolithic farmers (NFs). Unexpectedly, the change in the oral microbiome in the NFs seems to be stable and representative also in the medieval farmers’ (MFs) dental calculus, but the MHs carry dominantly cariogenic bacteria, such as *S. mutans* and show, in general, notably less diversity in the oral cavity*.* The authors reached these conclusions by showing that the abundance of Clostridiales and Ruminococcaceae was predictive for the HG groups compared to early NFs, and that the NFs and MFs had more periodontal disease-associated *P. Gingivalis, Tannerella* and *Treponema* generas. NFs, on the other hand could be differentiated from the MFs by different frequencies of *Veillonellaceae, Lachnospiraceae* and *Actinomucetales*. Lastly, the appearance of *S. mutans* in a high frequency occured only in the post-industrial MHs.

The evolution of commensal microbiota is still poorly understood, and the importance of the study by Adler *et al.* is not only limited to the archaeological interest of dietary questions in early human populations. Rather, this knowledge is of utmost importance for today’s healthcare, since balanced oral flora is crucial not only in the promotion of oral hygiene and the prevention of oral diseases (caries and periodontal diseases) but also in the prevention of the burdening chronic systemic diseases, such as arthritis, cardiovascular diseases, obesity and diabetes.

## Competing interests

The author has no competing interests.
